# Smoking and Socio-demographic correlates of BMI

**DOI:** 10.1186/s12889-016-3182-y

**Published:** 2016-06-10

**Authors:** Peizhi Wang, Edimansyah Abdin, Rajeswari Sambasivam, Siow Ann Chong, Janhavi Ajit Vaingankar, Mythily Subramaniam

**Affiliations:** Research Division, Institute of Mental Health, Buangkok Green Medical Park, 10 Buangkok View, Singapore, 539747 Singapore

**Keywords:** Obesity, Smoking, Health, Ethnicity

## Abstract

**Background:**

The aim of the current study was to examine the associations between Body Mass Index (BMI) and socio-demographic factors and to examine the relationship between BMI, smoking status and ethnicity.

**Methods:**

The Singapore Mental Health Study (SMHS) surveyed Singapore Residents (Singapore Citizens and Permanent Residents) aged 18 years old and above. BMI was calculated using height and weight which were self-reported by respondents. Socio-demographic characteristics and smoking status were recorded in a standardized data collection form.

**Results:**

Six thousand and six hundred sixteen respondents completed the study (response rate of 75.9 %) which constituted a representative sample of the adult resident population in Singapore. Ethnicity, gender and education status were associated with obesity. There was an interaction effect between ethnicity smoking status, and BMI. Indian and Malay smokers were less likely to be obese compared to Chinese smokers. The relationship between ethnicity and BMI was thus reversed when smoking was taken into account.

**Conclusions:**

The study identified certain subgroups and risk factors that are associated with obesity. There is a need for further research to explore and identify genetic, metabolic and ethnic differences that underlie the interaction between ethnicity and smoking status which affects BMI.

## Background

Smoking and obesity are the leading causes of preventable death [1]. Obesity significantly increases the risk of chronic diseases such as cardiovascular disease, musculoskeletal disorders, type- 2 diabetes, coronary heart disease, osteo-arthritis and certain cancers [2–6]. Every year, around 3.4 million adults die as a result of being overweight or obese [6]. Global estimates of disease burden attributed to overweight and obesity are substantial, with data indicating 7 % to 44 % [6]. A study in United States reported that the total cost attributed to obesity was US$99.2 billion dollars [7]. While no figures were reported for Singapore, WHO estimated that 2–7 % of total health care costs are attributed to obesity in developed countries [8].

Overweight and obesity are recognized as primary risk factors for chronic non-communicable disease (NCD) in Singapore [9]. These NCDs include Cancer (19 %), Cardiovascular Disease (20 %) and Diabetes Mellitus (10 %), all of which constitute about 195,840 life years lost due to mortality and ill-health in 2010 [9]. There is evidence that suggest that nicotine addiction influences body weight as well [10] and that a parabolic or U shaped relationship exists between BMI and smoking [11–13].

The consumption of nicotine products has been increasing worldwide. World Health Organization (WHO) has estimated that tobacco contributes 6 million deaths annually and is one of the most important causes of morbidity [14]. Nicotine is the key chemical compound that causes and sustains cigarette addiction and the design and contents of tobacco products today have made them more addictive than ever [15]. Smoking has been known to be linked to cancer, heart disease, stroke and other diseases [14]. However, the adverse effects of smoking are not limited to only the smoker; individuals who are exposed to second hand smoke are at an even greater risk for lung cancer, heart diseases and other chronic health problems [14]. Thus, at a societal level, smoking has substantial direct and indirect cost that puts a strain on health care and social resources.

Like many countries worldwide, smoking and obesity are serious public health concerns in Singapore. According to the Singapore National Health Survey conducted in 2004, there has been an increasing trend of smoking and obesity [16]. Prevalence rates for smoking have increased substantially from 6.9 % to 10.8 % from 2004 to 2010 [16, 17]. Similarly, prevalence rates for obesity have steadily increased from 12.6 % to 14.3 %, over the same period of time. BMI and fat rate have been observed to decrease with increased smoking intensity but to a certain point - should smoking intensity increase beyond a certain point, BMI is expected to increase exponentially [18].

Given the ethnic diversity in Singapore, an association between ethnicity and obesity has been previously documented. Studies have reported that people with Malay or Indian ethnicity have a high risk of being overweight or obese [17, 19]. Specifically, obesity was more common among Malays (24.0 %) followed by Indians (16.9 %) then Chinese (7.9 %) [17]. In the same study, the authors reported Malays had the highest daily smoking prevalence among the three ethnic groups. This brings forward a few uncertainties. Are the effects of nicotine directly influencing the risk of obesity? Does ethnicity alter the relationship between obesity and nicotine? The distinction is important because the latter would suggest that efforts to reduce smoking may be particularly important in reducing burden of obesity in some populations.

The aim of the current study was to examine the associations of BMI with socio-demographic factors and smoking in the Singapore resident population. We postulated that smokers would be less likely to be overweight and obese and that there is an interaction between smoking status and ethnicity which would affect BMI.

## Methods

### Subjects

The data for the current study was extracted from the Singapore Mental Health Study (SMHS) which was conducted from December 2009 to December 2010. This was a population-based, cross sectional, epidemiological study which has been described previously in greater detail [20]. A total of 6616 Singapore residents (including Singapore citizen and Permanent Residents) aged 18 years old and above were recruited. Written informed consent was obtained from all respondents and for those under the age of 21, consent was also obtained from a parent/legally acceptable representative. The study was approved by the relevant Institutional Review Boards.

### Assessments

The screening module of the Composite International Diagnostic Interview version 3.0 (CIDI 3.0) was used to assess smoking status. Respondents were asked whether they are current smokers, ex-smokers or non-smokers who have never smoked before. Ex-smokers and non-smokers were grouped together for analysis. Socio-demographic information was recorded in a standardized data collection form. BMI was calculated using height and weight which were self-reported by respondents. Participants were classified into four categories according to WHO International classification: (1) Underweight (≤18.5 kg/m^2^) (2) Normal Weight (18.5 kg/m^2^ to 24.9 kg/m^2^) (3) Overweight 25.0 kg/m^2^ -30.0 kg/m^2^) (4) Obese (≥30.0 kg/m^2^) [8].

### Statistical analysis

All estimates were weighted to adjust for oversampling and post-stratification sampling for age and ethnic distributions between the survey and the Singapore resident population. Weighted mean and standard error were calculated for continuous variables, and weighted percentages and standard errors for categorical variables. Multinomial regression models were used to generate odds ratio (ORs) and 95 % confidence intervals (CIs) using BMI as the main outcome variable and socio-demographic characteristics (i.e. age group, gender, ethnicity, marital status, employment status, income) and smoking status as predictor variables. Standard errors (S.E.) and significance tests were estimated using the Taylor series linearization method. All statistical analysis was carried out using Statistical Analysis Software (SAS) System version 9.2. Statistical significance was evaluated at the <0.05 level using two-sided tests.

## Results

In all, 6616 face-to-face interviews were completed, yielding a response rate of 75.9 %. Due to missing data in a small number of cases, BMI could only be calculated for 6291 (48.9 % male and 51.1 % female) respondents. The mean (SD) age of the sample was 42 (14.5) years.

Table 1 presents the socio-demographic distribution of the study sample. 4.7 % of the study sample had an education level of primary school and below, 14.1 % had secondary school education, 27.9 % had Pre-U/junior college/diploma, 22.9 % had vocational training and 8.0 % had university education. Approximately 23.6 % was overweight and 7.8 % was obese. 16.3 % of the population reported being a current smoker and 83.7 % of the population were non-smokers at the point of recruitment.

**Table 1:** Socio-demographic distribution of the sample

	Number	Weighted %	S.E.
Age group			
18–34	2264	32.4	-
35–49	2308	34.7	-
50–64	1401	22.6	-
65+	318	10.4	-
Ethnicity			
Chinese	1976	77.9	-
Malay	2145	11.4	-
Indian	1905	8.2	-
Others	265	2.5	-
Sex			
Male	3191	48.9	0.9
Female	3100	51.1	0.9
Marital			
Single	1787	29.4	0.7
Married	4076	62.4	0.8
Divorced/separated	248	4.2	0.4
Widowed	178	3.9	0.4
Education			
Primary and below	203	4.7	0.4
Secondary	813	14.1	0.6
Pre-u/junior-college/diploma	1905	27.9	0.8
Vocational	1331	22.9	0.7
University	706	8.0	0.4
Employment			
Employed	4464	71.8	0.8
Economically inactive^a^	1365	23.8	0.7
Unemployed	285	4.4	0.4
Income			
Below $SD 20,000	3110	50.3	0.9
$SD 20,000–49,999	1900	31.8	0.8
Above $SD 50,000	955	17.9	0.7
BMI			
Underweight	446	8.4	0.5
Normal	3338	60.3	0.9
Overweight	1719	23.6	0.8
Obese	788	7.8	0.4
Smoking			
No	4891	83.7	0.6
Yes	1270	16.3	0.6

^a^Include homemakers, students and retirees/pensioners

Table 2 shows the socio-demographic correlates among three groups: Obese vs. Normal, Overweight vs. Normal and Underweight vs. Normal among the study population. Among the different ethnicities in Singapore, Malays were more likely to be obese (OR = 4.7) and overweight (OR = 2.0) as compared to the Chinese. Indians were also more likely to be obese (OR = 3.6) and overweight (OR =2.0) as compared to the Chinese. Indians were 0.6 times less likely to be underweight than the Chinese. Other ethnicities were also 2.7 times more likely to be obese compared to Chinese. Females were less likely to be obese (OR = 0.7) and more likely to be underweight (OR = 2.8), respectively. Those who were obese were more likely to have lower education [primary and below (OR = 2.1), secondary (OR = 1.8), pre-U/Junior College/Diploma (OR = 1.8) and Vocational (OR = 2.3)].

**Table 2:** Socio-demographic correlates of Body Mass Index (BMI)

		Obese vs			Overweight vs			Underweight vs		
		Normal weight			Normal weight			Normal weight		
		OR^+^	95 % CI	*P* value	OR^+^	95 % CI	*P* value	OR^+^	95 % CI	*P* value
Age group (years)	18 to 34	Ref			Ref			Ref		
	35 to 49	1.0	(0.7–1.6)	.87	1.2	(0.9–1.5)	.29	0.6	(0.4–0.9)	**.0087**
	50 to 64	1.2	(0.8–2.0)	.3739	1.3	(1.0–1.8)	.0599	0.5	(0.3–0.9)	**.0148**
	65 and above	0.4	(0.2–1.0)	0.0611	1.4	(0.8–2.3)	.2548	0.6	(0.2–1.6)	.3066
Ethnicity	Chinese	Ref								
	Malay	4.7	(3.6–6.2)	**<.0001**	2.0	(1.7–2.5)	**<.0001**	0.7	(0.5–1.0)	.0539
	Indian	3.6	(2.7–4.7)	**<.0001**	2.0	(1.7–2.4)	**<.0001**	0.6	(0.4–0.8)	**.0025**
	Others	2.7	(1.5–4.9)	**.0016**	1.4	(0.9–2.0)	.0974	0.4	(0.2–1.3)	.1319
Sex	Male	Ref								
	Female	0.7	(0.5–0.9)	**.0127**	0.6	(0.5–0.7)	**<.0001**	2.8	(2.0–4.0)	**<.0001**
Marital	Single	Ref								
	Married	1.3	(0.8–2.0)	.2887	1.2	(1.0–1.6)	.1209	0.6	(0.5–0.9)	**.0134**
	Divorced/Separated	1.4	(0.6–3.1)	.4468	0.8	(0.5–1.4)	.3988	0.8	(0.4–1.7)	.5659
	Widowed	2.1	(0.8–5.6)	.1611	1.6	(0.8–3.3)	.2361	0.7	(0.2–2.4)	.5974
Education	Primary and below	2.1	(1.2–3.9)	**.0123**	1.4	(0.9–2.1)	.0976	1.0	(0.5–2.0)	.9412
	Secondary	1.8	(1.1–2.9)	**.0208**	1.3	(0.9–1.7)	.167	1.1	(0.7–1.8)	.7876
	Pre-U/Junior-college/Diploma	1.8	(1.1–2.9)	**.0262**	1.1	(0.8–1.5)	.4815	0.9	(0.6–1.4)	.7431
	Vocational	2.3	(1.3–4.1)	**.0058**	1.1	(0.7–1.6)	0.6593	1.6	(0.9–2.7)	.1257
	University	Ref								
Employment	Employed	Ref								
	Economically inactive^#^	1.1	(0.7–1.5)	.7408	0.8	(0.6–1.1)	.1888	0.9	(0.6–1.3)	.4997
	Unemployed	1.3	(0.7–2.4)	.4012	0.8	(0.5–1.4)	.401	1.2	(0.7–2.1)	.6006
Income	Below $SD 20,000	Ref								
	$SD 20,000 to 49,999	1.2	(0.9–1.7)	.2873	1.0	(0.8–1.3)	.8953	0.7	(0.5–1.1)	.1109
	$SD 50,000 and above	0.9	(0.5–1.5)	.6098	1.1	(0.8–1.6)	.4576	0.6	(0.4–1.1)	.1117
Smoking	No	Ref								
	Yes	1.5	(0.9–2.5)	.1411	1.1	(0.8–1.6)	.5584	1.0	(0.6–1.8)	.9424

Bold font indicates significant *p* values

^#^ includes retirees, students and housewives

+Odds Ratios derived from multinomial regression

Ref: Reference

It was observed that there was no significant effect on weight when smoking status was taken into account. We further explored a possible interaction of each significant variable in the model. We found there was an interaction effect between ethnicity and smoking status, affecting BMI. Indian smokers were less likely to be obese and more likely to be underweight compared to Chinese smokers (OR = 0.4, CI 0.2–0.8, *p* = .0116 and OR = 2.8, CI 1.4–5.9, *p* = .0048 respectively). Similarly, Malay smokers were less likely to be obese and overweight (OR = 0.3, CI 0.2–0.5, *p* < .0001 and OR = 0.6, CI 0.4–0.9, *p* = 0.0072 respectively) than Chinese smokers.

## Discussion

This study has allowed us to look at the correlates of overweight, obesity and underweight with socio-demographic factors in a multi-ethnic Asian population. Results indicate that ethnicity plays a significant role in obesity with the Malays and Indians more likely to be overweight and obese when compared to the Chinese. This finding is in accordance with the National Health Survey conducted in 2010 [17]. This relationship was however, reversed when smoking was taken into account. Indian and Malay smokers were at a lesser at risk of being overweight and obese than Chinese smokers.

Analysis also revealed that the odds of obesity and overweight do not increase with age. This finding is in contrast to a previous study, whereby the authors reported that the odds of obesity and overweight increases with age and it is most prevalent among the middle aged individuals (50–59 years old) [8]. Unlike the previous study where underweight groups were not looked at, the current findings found that adults aged 35–49 years old (OR = 0.6) and 50–64 years old (OR = 0.5) were less likely to be underweight as compared to the young adults (18–34 years old).

The National Health Survey Singapore 2010 reported that the prevalence of obesity was highest among the Malay (38.0 %) followed by the Indians (32.8 %) and Chinese (19.4 %) [17]. Our findings suggest that the Malays had the highest odds of being obese, followed by the Indians. Ethnic differences in susceptibility to obesity have also been observed overseas [21]. However, it is unclear whether the effects of weight are directly linked to the biological composition of the different ethnic groups or other various reasons, i.e. food intake and/or physical activity. The mechanisms underlying the body weight differences among the ethnic groups are unclear. One possibility which had been suggested was that individuals from different ethnic groups have a differing propensity to accumulate fats in regions that are detrimental, such as within the abdomen, in the skeletal muscles or liver [22].

As expected, our findings on gender differences show that females were significantly more likely to be underweight as compared to males. This result correspond with the National Health Survey 2010, which reported a similar trend of males (12.1 %) being more likely to be obese when compared to females (9.5 %) [17]. Anecdotal evidence suggests that it is acceptable for men to be overweight, but the same may not apply to females. Overweight females tend to be stigmatized while underweight are encouraged and valued in western influenced countries, such as Singapore [23]. This can be also be reflected by the difference in body esteem reported between the genders, whereby girls are more vulnerable to negative evaluation of being overweight than boys [24].

Surprisingly, there was no significant association between marital status and obesity/overweight. This was contrary to previous findings which reported married individuals are at an increased risk of being overweight [19]. Our findings instead suggest that marital status was a protective factor for being underweight. An increase in weight after getting married has been observed elsewhere [25, 26]. Studies have suggested that there are role changes for married couples. Individualistic activities such as exercise take a backseat to marital obligations. People who are married are also less likely to smoke and more likely to quit smoking [25]. Anecdotal evidence suggests that children could also influence the parents’ eating patterns, with the parent eating the leftovers to minimize wastage. All of these factors may lead to weight gain.

We also examined the socioeconomic factors which predispose people to be overweight and obese. Previous studies have suggested that socioeconomic factors are one of the most consistent predictors of body weight [27, 28]. Socioeconomic factors such as education, income and occupation are related to variations in behavior which changes energy intake, expenditure and metabolism [29]. We found that obesity was inversely associated with education level, with lower education linked to higher risk of obesity. Education has been hypothesized to enable people to integrate healthy behaviors (e.g., dietary choices, nutrition, access to healthy food, exercise) into a lifestyle, which gives them a sense of control over their health [30]. It also limits one's exposure to negative influences associated with the social environment in which one lives and works [30]. An individual with higher education is likely to have the means to access the various avenues that help maintain a healthy life study including gym membership and sporting clubs.

Although the general consensus is that smoking status is negatively associated with BMI [10, 18], and our study results did not show a significant inverse relationship between smoking status and BMI. This could be due to the contribution of external factors such as eating habits and lifestyle habits such as amount of exercise. However, when smoking was taken into account, the effect of ethnicity on BMI was reversed. Indian and Malay smokers were less likely to be obese when compared to Chinese smokers. Multiple factors may come into play that could affect an individual’s body weight. Considerations such as energy intake, energy expenditure and lifestyle may contribute to the inverse association.

In previous research conducted in Singapore, distinct ethnic differences associated with nicotine dependence were found. Malays were reported to have the highest prevalence of nicotine dependence compared to Indian and Chinese [31, 32]. Taking this into considerations, it is plausible that nicotine acts as an appetite suppressant, thus effectively reducing the calories consumption for smokers. In terms of physical activity, Malays tend to have the highest levels, followed by Indians which suggest differing higher energy expenditure among the three ethnic groups [33]. Some other studies, have instead suggested that smoking is associated with energy expenditure, with resting energy expenditure differing between ethnicities [34]. To the best of our knowledge, literature concerning the relationship of smoking and energy expenditure among the three ethnic groups has not been looked at in Singapore. Our findings highlight that further research is needed to examine the interaction between smoking and other socio-demographic factors.

### Strengths and limitations

The study has some limitations. This study was conducted via face to face household survey. Thus, information from residents of nursing homes, hospitals and prisons for the entire field period of the survey was excluded. About 24 % of the sample was not interviewed due to “non-response”. This non-response rate could lead to underestimation of the true rates. Parameters collected, such as height and weight were self-reported. While previous studies have suggested that there is a high correlation between self-reported and measured parameters [35]. Others have suggested that self-report may underestimate weight and overestimate height [36, 37]. Abdominal fatness was not measured or examined in this study. Previous studies have suggested central obesity such as waist circumference, waist-hip ratio and waist-stature ratio is a better indicator for risk of cardiovascular heart disease, diabetes and hypertension for Asians [38–40]. However, BMI is an equally strong indicator [39]. Past smoking behavior and intensity of smoking was not looked at in this study, which could have affected the individual body weight.

Lastly, due to the cross sectional design of the study, we are not able to attribute any casual relationships between the relationship of BMI and smoking. Despite these limitations, our study includes a large sample size with a reasonable response rate which makes it a good representative study of the adult population in Singapore. The study was also a single phase study without geographical clustering that ensured that detailed data was collected from all individuals across the island.

## Conclusion

In summary, our study identified certain subgroups and risk factors that are associated with obesity. We observed an interaction effect between ethnicity smoking status, and BMI. There is a need for further research to explore and identify genetic, metabolic and ethnic differences that underlie the interaction between ethnicity and smoking status which affects BMI.

## Abbreviations

BMI, body mass index; CI, confidence interval; CIDI, Composite International Diagnostic Interview; NCD, non-communicable disease; OR, odds ratio; S.E., standard error; SAS, statistical analysis software; SD, standard deviation; SMHS, Singapore Mental Health Study; WHO, World Health Organization.
